# Astrocyte ryanodine receptors facilitate gliotransmission and astroglial modulation of synaptic plasticity

**DOI:** 10.3389/fncel.2024.1382010

**Published:** 2024-05-14

**Authors:** Ulyana Lalo, Yuriy Pankratov

**Affiliations:** School of Life Sciences, University of Warwick, Coventry, United Kingdom

**Keywords:** glia-neuron interaction, caffein, aging, microdomain Ca^2+^ activity, glutamate release, ATP release

## Abstract

Intracellular Ca^2+^-signaling in astrocytes is instrumental for their brain “housekeeping” role and astroglial control of synaptic plasticity. An important source for elevating the cytosolic Ca^2+^ level in astrocytes is a release from endoplasmic reticulum which can be triggered via two fundamental pathways: IP3 receptors and calcium-induced calcium release (CICR) mediated by Ca^2+^-sensitive ryanodine receptors (RyRs). While the physiological role for glial IP3 became a focus of intensive research and debate, ryanodine receptors received much less attention. We explored the role for ryanodine receptors in the modulation of cytosolic Ca^2+^-signaling in the cortical and hippocampal astrocytes, astrocyte-neuron communication and astroglia modulation of synaptic plasticity. Our data show that RyR-mediated Ca^2+^-induced Ca^2+^-release from ER brings substantial contribution into signaling in the functional microdomains hippocampal and neocortical astrocytes. Furthermore, RyR-mediated CICR activated the release of ATP and glutamate from hippocampal and neocortical astrocytes which, in turn, elicited transient purinergic and tonic glutamatergic currents in the neighboring pyramidal neurons. The CICR-facilitated release of ATP and glutamate was inhibited after intracellular perfusion of astrocytes with ryanodine and BAPTA and in the transgenic dnSNARE mice with impaired astroglial exocytosis. We also found out that RyR-mediated amplification of astrocytic Ca^2+^-signaling enhanced the long-term synaptic potentiation in the hippocampus and neocortex of aged mice. Combined, our data demonstrate that ryanodine receptors are essential for astrocytic Ca^2+^-signaling and efficient astrocyte-neuron communications. The RyR-mediated CICR contributes to astrocytic control of synaptic plasticity and can underlie, at least partially, neuroprotective and cognitive effects of caffein.

## Introduction

Astrocytes are an instrumental component of brain cellular networks. Apart from generally acknowledged brain “housekeeping” functions, such as metabolic and structural support of neurons and control of neuro-vascular interface, astrocytes have recently been implicated into higher brain functions, like information processing and learning and memory (Adamsky et al., 2018; Covelo and Araque, 2018; Savtchouk and Volterra, 2018; Durkee and Araque, 2019; Durkee et al., 2021; Lalo et al., 2021; Koh et al., 2022). Participation of astrocytes in brain computation is underlined by their capability to receive and integrate information on activity of large ensembles of synapses (Giaume et al., 2010; Halassa and Haydon, 2010; Bazargani and Attwell, 2016; Savtchouk and Volterra, 2018; Lalo et al., 2021) and communicate back to neurons via release of small molecule transmitters (gliotransmitters) such as ATP, glutamate and D-Serine (Araque et al., 2014; Lalo et al., 2014, 2021; Savtchouk and Volterra, 2018; Durkee and Araque, 2019; Koh et al., 2022; Lalo and Pankratov, 2023). The integrative function of astroglia and, thereby, efficient glia-neuron communication, critically depends on the elevation of cytosolic Ca^2+^ in astrocytes which is necessary to activate various pathways of gliotransmission (Araque et al., 2014; Bazargani and Attwell, 2016; Savtchouk and Volterra, 2018; Durkee and Araque, 2019; Durkee et al., 2021; Lalo et al., 2021). Astrocytic Ca^2+^-signaling can also mediate activity-dependent modulation of their “housekeeping” functions (Gourine et al., 2010; Halassa and Haydon, 2010; Bazargani and Attwell, 2016).

Although mechanistic details of Ca^2+^-dynamics in astrocyte, in particular in different subcellular compartments, and their relevance for astroglial modulation of synaptic plasticity and cognitive function are still being elucidated, a pivotal role for Ca^2+^-signaling in astrocyte physiology is commonly accepted (Bazargani and Attwell, 2016; Savtchouk and Volterra, 2018; Verkhratsky and Nedergaard, 2018). There is also emerging evidence that impairment of astroglial Ca^2+^ signaling can contribute to various psychiatric and neurodegenerative disorders and age-related cognitive decline (Liu et al., 2017; Lalo et al., 2018; Morita et al., 2019; Verkhratsky et al., 2019; Lalo and Pankratov, 2021).

The main sources of elevating the cytosolic Ca^2+^ level in astrocytes include release from intracellular stores of endoplasmic reticulum (ER), release from mitochondria and entry via plasma membrane ligand-gated Ca^2+^ channels (Reyes and Parpura, 2009; Palygin et al., 2010; Rasooli-Nejad et al., 2014; Bazargani and Attwell, 2016; Agarwal et al., 2017; King et al., 2020). The release from ER-stores possesses two fundamental activation pathways: via IP3 receptors and calcium-induced calcium release (CICR) mediated by Ca^2+^-sensitive ryanodine receptors (RyRs) (Berridge, 2016). During last two decades of research into specific mechanisms of Ca^2+^-signaling in astrocytes, focus was made on role for IP3 receptors (Bazargani and Attwell, 2016; Savtchouk and Volterra, 2018; King et al., 2020). To a large extent, this was underlined by the debate on impact of astroglia-specific deletion of IP3 receptors on glia-neuron interactions and synaptic plasticity.

At the same time, an alternative mechanism of activation of Ca^2+^-release from ER– via RyRs – remained underexplored. Although earlier studies (Golovina and Blaustein, 1997; Simpson et al., 1998; Hua et al., 2004) suggested the participation of RyRs in generation of Ca2 + -transients and Ca2 + -waves in astrocytes, the physiological importance of RyRs for brain function was traditionally associated with neuronal and vascular cells. In these cells, RyRs are usually activated by Ca2 + influx via voltage-gated Ca^2+^ channels, and lack of such channels in astroglial cells undermined, in common opinion, a significance of RyRs for glia signaling and glia-neuron communications. However, in astrocytes, an initial influx of Ca^2+^- to trigger a substantial RyR-mediated Ca^2+^ release from ER could be provided by ionotropic NMDAR and P2X receptors and SOC channels (Reyes and Parpura, 2009; Palygin et al., 2010; Weiss et al., 2022).

Nowadays, interest to the role for RyRs in the glial function is growing and evidence of their participation in the astrocytic signaling is accumulating (Reyes and Parpura, 2009; Pankratov and Lalo, 2015; Skowronska et al., 2020; Weiss et al., 2022). In our previous work, we have demonstrated that RyRs can bring a substantial contribution into Ca^2+^-transients triggered in neocortical astrocytes by noradrenaline (Pankratov and Lalo, 2015). The recent data of Weiss et al. (2022) suggest that RyR-mediated Ca^2+^ release from ER takes part in the propagation of Ca^2+^ waves between neighboring glial cells and that this mechanism plays important role in maintaining normal brain excitability. So, a putative amplification of astrocytic Ca^2+^-signaling by RyR-dependent CICR might have important implications for glia-neuron interactions which are yet to be fully understood.

The putative action of RyR as an “enhancer” of astrocytic signaling can gain a special importance in the aging brain. There is accumulating reports that age-related astrocyte atrophy and dysfunction, in particular the decrease in Ca^2+^-signaling, can underlie neurodegeneration and impairment of synaptic transmission and plasticity (Lalo et al., 2011, 2020; Sadick and Liddelow, 2019; Siracusa et al., 2019; Verkhratsky et al., 2019; Lalo and Pankratov, 2021). At the same time, manipulations that enhance astrocytic Ca^2+^-signaling, such as application of exogenous noradrenaline or changes in lifestyle and diet, can ameliorate the age-related decline in cognitive functions (Lalo et al., 2019, 2020; Wahl et al., 2019; Lalo and Pankratov, 2021). In line with this notion, there are multiple evidence of beneficial effects of RyR agonist caffein on the function of aging brain (Eskelinen and Kivipelto, 2010; Santos et al., 2010; Barberger-Gateau et al., 2013) including amelioration of decline in learning and memory in the AD model mice (Arendash et al., 2006; Laurent et al., 2014; Stazi et al., 2023). Still, the role of activation of RyRs in the cognitive effects of caffein in aging brain remains unexplored.

In the present paper, we explore the role for ryanodine receptors in the modulation of cytosolic Ca^2+^-signaling in the cortical and hippocampal astrocytes and astrocyte-driven modulation of synaptic plasticity across a lifetime. To dissect the impact of astrocytic RyRs on synaptic signaling and plasticity, we compared the effects of RyRs modulators in the wild-type and dnSNARE transgenic mice with impaired gliotransmission (Halassa et al., 2009; Lalo et al., 2014, 2016) of two age groups.

## Materials and methods

All animal work has been carried out in accordance with UK legislation and “3R” strategy; research did not involve non-human primates. This project was approved by the University of Warwick Animal Welfare and Ethical Review Body (AWERB), approval number G13-19, and regulated under the auspices of the UK Home Office Animals (Scientific Procedures) Act licenses P1D8E11D6 and I3EBF4DB9. Experiments were carried out in the astrocytes and neurons of the hippocampus and somatosensory cortex of dnSNARE transgenic mice (Halassa et al., 2009; Lalo et al., 2014) and their wild-type littermates (WT) of two aged groups, 2.5 – 4.5 (average 3.6) months and 14–18 (average 16.1) months; these groups were referred correspondingly as adult and old. In some experiments, the transgenic mice expressing enhanced green fluorescent protein (EGFP) under the control of the glial fibrillary acidic protein (GFAP) promoter (GFAP-EGFP) were used (Palygin et al., 2010; Lalo et al., 2011; Rasooli-Nejad et al., 2014). For clarity, all data referred here as wild-type are reported solely for wild-type littermates to dnSNARE mice; usage of GFAP-EGFP mice was explicitly stated where appropriate.

### Slice and cell preparation

Mice were anesthetized by halothane and then decapitated; brains were rapidly removed and placed into ice-cold physiological saline containing (mM): NaCl 130, KCl 3, CaCl_2_ 0.5, MgCl_2_ 2.5, NaH_2_PO_4_ 1, NaHCO_3_ 25, glucose 15, pH of 7.4 gassed with 95% O_2_ - 5% CO_2_. Transverse slices (280 μm) were cut at 4^°^C and then placed in physiological saline containing (mM): NaCl 130, KCl 3, CaCl_2_ 2.5, MgCl_2_ 1, NaH_2_PO_4_ 1, NaHCO_3_ 22, glucose 15, pH of 7.4 gassed with 95% O_2_ - 5% CO_2_ and kept for 1 - 5 h prior to cell isolation and recording. The same extracellular saline was used for the recordings with addition of 1 μM TTX except experiments that required the evoking synaptic activity, namely: registration of astrocytic Ca^2+^-signaling elicited by stimulation of neuronal afferents and long-term plasticity experiments (detailed below).

### Electrophysiological recordings

Whole-cell voltage-clamp recordings from the CA1 and neocortical neurones were performed using glass patch-pipettes (4 - 5 MΩ) filled with intracellular solution (in mM): 110 CsCl, 10 NaCl, 10 HEPES, 5 MgATP, 1 D-Serine, 0.1 EGTA, pH 7.35; Transmembrane currents were monitored using an MultiClamp 700B patch-clamp amplifier and Digidata1440A data acquisition board (Axon Instruments, USA), filtered at 2 kHz and digitized at 4 kHz. Experiments were controlled by WinWCP software; recordings were analyzed using self-designed software. Liquid junction potentials were compensated with the patch-clamp amplifier. The series and input resistances were respectively 5–7 MΩ and 600–1100 MΩ; both series and input resistance varied by less than 20% in the cells accepted for analysis.

In the synaptic plasticity experiments, the same slice preparations were used. Field excitatory postsynaptic potentials (fEPSPs) were measured via a glass micropipette filled with extracellular solution (0.5 - 1 MΩ resistance) placed in neocortical layer 2/3 or in the *stratum radiatum* of the CA1 area. The fEPSPs were evoked correspondingly by the stimulation of neuronal afferents descending from layers IV-V or stimulation of CA3-CA1 Schaffer collaterals with a bipolar coaxial electrode (WPI, Stevenage, UK); stimulus duration was 300 μs, the stimulus strength was set to provide average the fEPSP amplitude about 30–40% of maximal level (typically 1.5–3 μA in neocortex and 1.2–2.5 μA in hippocampus).

To induce the long-term potentiation (LTP), several of trains of high-frequency theta-burst stimulation (HFS-trains) have been delivered; each HSF-train consisted of 10 pulses stimulated at 100 Hz (100 ms total length), trains were delivered with 200 ms interval and every 10 trains were separated by 10 s-long intervals. For the weak and strong stimulation of CA3-CA1 synapses correspondingly 5 and 10 trains were used. In the L2/3 area, the weak and strong stimulation consisted correspondingly of 20 and 50 trains.

### Multi-photon fluorescent Ca^2+^-imaging

To evaluate the intracellular concentration of free Ca^2+^ ([Ca^2+^]_i_) in CA1 and layer 2/3 astrocytes *in situ*, brain slices were incubated with 1 μM of Rhod-2AM for 30 min at 33^°^C. Usage of relatively short incubation time ensured preferential loading of astrocytes (as compared to neurons) with Ca^2+^-dye, as reported previously (Perea and Araque, 2005; Rasooli-Nejad et al., 2014) Astrocytes were identified initially by their morphology under DIC observation and EGFP fluorescence (astrocytes from dnSNARE mice and GFAP-EGFP mice). After the recording identification of astrocytes was confirmed by their electrophysiological properties as described previously (Lalo et al., 2014; Rasooli-Nejad et al., 2014; Pankratov and Lalo, 2015).

Two-photon images of neurons and astrocytes were acquired at 5Hz frame-rate using a Zeiss LSM-7MP multi-photon microscope coupled to a SpectraPhysics MaiTai pulsing laser; experiments were controlled by ZEN LSM software (Carl Zeiss, Jena, Germany). Images were analyzed offline with aid of ZEN LSM (Carl Zeiss) and ImageJ 1.52 (NIH) software. The [Ca^2+^]_i_ levels were expressed as ΔF/F ratio averaged over a region of interest (ROI). To analyze spontaneous Ca^2+^–signaling in astrocytes, several ROIs located over peripheral astrocytic processes (functional microdomains) and 1 ROI located over the soma were chosen. The initial identification of peripheral ROIs as microdomains was performed with the aid of standard particle analysis routine of ImageJ. After then, identification of microdomains was verified by quantification of relative fluorescence time course. Only regions, exhibiting average fluorescence 3 times larger than standard deviation of noise for the period of 1 s at least once over 30 min, were selected as active microdomains for further analysis. The average frequency and amplitude of spontaneous Ca^2+^-transients were calculated for all ROIs detected in each cell (typically 10–15 ROIs per cell). To test the response of astrocyte to synaptic stimulation, a single HFS train (as describe above) was used. The net Ca^2+^ -response to agonists or synaptic stimulation was quantified using an ROI covering the whole cell image.

### Measurement of extracellular ATP concentration in brain tissue

The DHPG-induced changes in the ATP concentration in the neocortical and hippocampal tissue were evaluated with the aid of microelectrode biosensors produced by the Sarissa Biomedical (Coventry, UK). A detailed description of the biosensors and recording procedure has been published previously in Frenguelli et al. (2007). Briefly, biosensors consisted of the thin (25 μM) Pt/Ir wire coated with permselective polymer matrix with immobilized enzymes metabolizing ATP in a cascade of redox reactions, so the current output of the sensor was proportional to the ambient ATP concentration. To calculate the ATP concentration, the auxiliary null-sensor, coated with the matrix but containing no enzymes was inserted in the vicinity of main the ATP-sensor and its signal was subtracted from the signal of main sensor. Usage of null-sensor enabled to minimize a putative interference from any unspecific electro-active endogenous substances (Frenguelli et al., 2007). To further reduce an interference from unspecific signals, only transient elevation of the ATP-signal from baseline level was assessed. To calibrate the measurements and compensate for any reduction in sensitivity during experiment, biosensor response to the application of known concentrations of ATP (10 μM) was recorded twice in each experiment, before the slice was present in the perfusion chamber and after the slice had been removed.

### Data analysis

All data are presented as mean ± standard deviation (SD) and the statistical significance of differences between data groups was tested by two-tailed unpaired t-test, unless indicated otherwise. For all cases of statistical significance reported, the statistical power of the test was 0.8–0.9. Each brain slice was used only for one experiment (e.g., fluorescent recordings in single astrocyte or single LTP experiment either in CA1 or layer 2/3). The number of experiments/cells reported is therefore equal to the number of slices used. The experimental protocols were allocated randomly so the data in any group were drawn from at least from 3 animals, typically from 4 to 10 mice. The average ratio of experimental unit per animal was 1.5 for the LTP experiments and 1.4 whole-cell recordings and fluorescent Ca^2+^-measurements.

The spontaneous transmembrane currents recorded in neurons were analyzed off-line using methods described previously (Lalo et al., 2014, 2016). The amplitude distributions of spontaneous and evoked currents were analyzed with the aid of probability density functions and likelihood maximization techniques; all histograms shown were calculated as probability density functions.

### Drugs

Receptor antagonists and agonists (NMDA, D-AP5, NBQX, ryanodine, TFLLR, DPCPX, SCH58261) were from the Tocris (Bristol, UK). Other salts and chemicals (Rhod-2, Rhod-2AM, noradrenaline, caffeine, TTX) were from Sigma (Dorset, UK) unless specifically indicated.

## Results

### Impact of ryanodine receptor modulators on Ca^2+^-signaling in astrocytes

**FIGURE 1:**
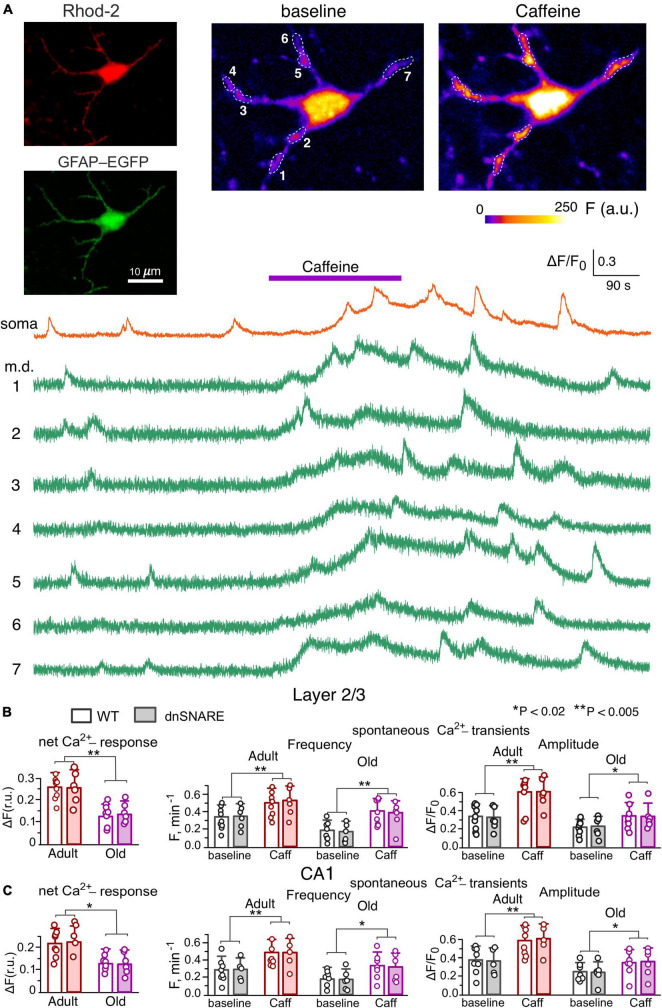
Caffein-induced Ca^2+^-signaling in neocortical and hippocampal astrocytes. **(A)** Representative multi-photon images of EGFP fluorescence and presudo-color images of Rhod-2 fluorescence recorded in the astrocyte of the dnSNARE mouse before (baseline) and after the application of RyR agonist caffein. Examples of Ca^2+^-transients recorded in the soma and microdomain (m.d.) ROIs indicated in the images are shown below. Note the marked increase in the spontaneous Ca^2+^-elevations induced by application of caffein. **(B,C)** The pooled data on the net cell responses to caffein and the frequency and amplitude of spontaneous Ca^2+^- transients recorded in the layer 2/3 **(B)** and CA1 **(C)** astrocytes of WT and dnSNARE mice of two age groups. The data are shown as mean ± SD; dots indicate the data obtained in the individual astrocytes. Asterisks (*,**) indicate the statistical significance of effects of caffein. Note the lack of difference in the effect of caffein of Ca^2+^ -signaling in the WT and dnSNARE mice and responsiveness of CA1 and layer 2/3 astrocytes to RyR agonist.

**FIGURE 2:**
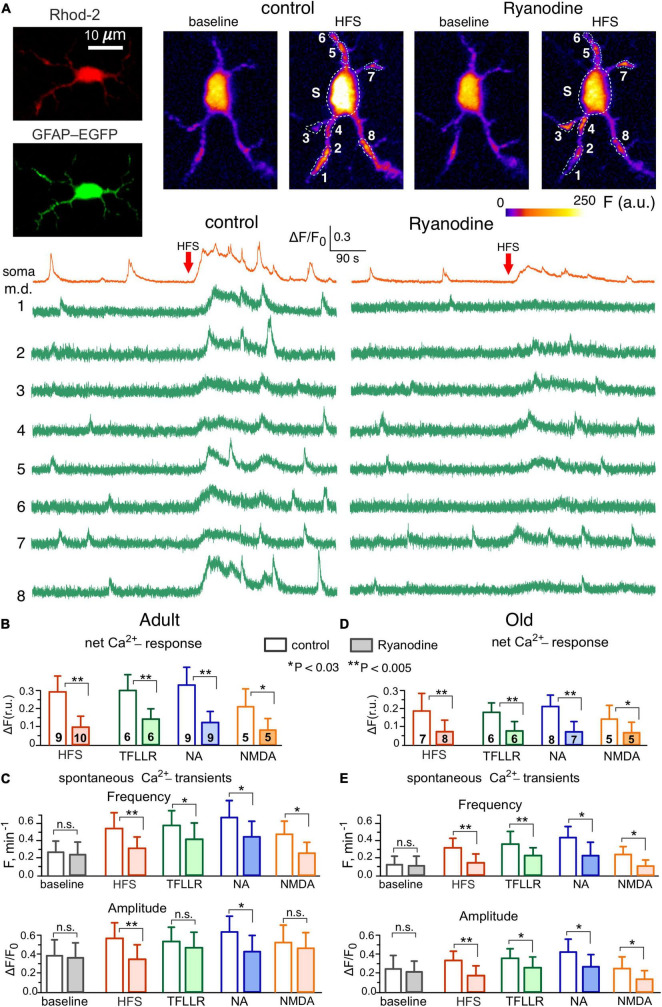
Contribution of Ryanodine receptors to Ca^2+^-signaling in the hippocampal astrocytes. **(A)** Representative multi-photon images of EGFP fluorescence and presudo-color images of Rhod-2 fluorescence recorded in the CA1 astrocyte of old dnSNARE mouse before (baseline) and after the short train of high-frequency stimulation of CA3-CA1 synapses (HFS). Examples of Ca^2+^-transients recorded in the soma and microdomain (m.d.) ROIs in control (left) and in the presence of 10 μM ryanodine (right) are shown below. Note the marked increase in the spontaneous Ca^2+^-elevations after HFS in control and inhibition of the HFS-induced signaling by ryanodine. **(B–E)** The pooled data on the net cell responses to HFS and agonists of PAR-1 (TFLLR, 10 μM), adrenergic (NA, 3 μM) and NMDA receptors **(B,D)** and the frequency and amplitude of the spontaneous Ca^2+^- transients **(C,E)** recorded in the CA1 astrocytes of adult **(B,C)** and old **(D,E)** mice in control and in presence of ryanodine. The data are shown as mean ± SD for the cell numbers indicated in panels **(B,D)**. Asterisks (*,**) indicate the statistical significance of the effects of ryanodine. Note the sensitivity of CA1 astrocytes to ryanodine and age-related in Ca^2+^-signaling.

We investigated the action of RyRs activator caffeine and selective inhibitor ryanodine (Zucchi and Ronca-Testoni, 1997) on the Ca^2+^-signaling in astrocytes of CA1 hippocampal area and neocortical layer 2/3 *in situ* (Figures 1, 2). We used the dnSNARE mice and their wild type littermates of two age groups, adult (2.5 – 4 months old) and old (14 – 18 months old).

We evaluated the spontaneous and evoked Ca^2+^-transients in the somata and functional microdomains (Shigetomi et al., 2013; Savtchouk and Volterra, 2018), located on the astrocytic branches (Figures 1A, 2A, also see Materials and methods). Under baseline conditions, neocortical and hippocampal astrocytes exhibited prominent spontaneous Ca^2+^-transients, whose amplitude and frequency showed notable decrease at the old age. In the layer 2/3 astrocytes, the average baseline frequency of spontaneous Ca^2+^-transients (pooled for the whole cell image) was 0.34 ± 0.15 min^–1^ in the young WT mice and 0.19 ± 0.08 min^–1^ in the old WT mice; the average relative amplitudes of Ca^2+^-transients were correspondingly 0.32 ± 0.13 and 0.21 ± 0.09 (Figures 1A, B). The spontaneous signaling in the layer 2/3 astrocytes of dnSNARE mice was not significantly different from their wild-type littermates (Figure 1B). The similar pattern of age-related changes and lack of difference between WT and dnSNARE mice was exhibited by the spontaneous Ca^2+^-signaling in the CA1 astrocytes (Figure 1C).

Bath application of 20 μM caffeine in the presence of A1 and A2 receptors antagonists (Bastia et al., 2002) DPCPX (3 μM) and SCH58261 (1 μM), caused a slow and sustainable elevation of the cytosolic Ca^2+^-level in cortical astrocytes of adult mice (Figure 1A) accompanied by the notable increase in the number of active microdomains and the amplitude and frequency of microdomain Ca^2+^-transients (Figures 1A, B). Whereas the integral Ca^2+^-response of cortical astrocytes to caffeine underwent a sharp decline with aging, the caffeine-induced augmentation of microdomain signaling was retained in the layer 2/3 astrocytes of old mice, both WT and dnSNARE (Figure 1B). The CA1 astrocytes showed similar behavior in the respect of caffeine-induced effects (Figure 1C). These data suggest that RyR-mediated release from ER can play a prominent role in the augmentation of astroglial Ca^2+^-signaling, both in adult and old age.

To verify the importance of RyR-mediated CICR for the astroglial signaling activated by release of various neurotransmitters, we evaluated the impact of ryanodine (10 μM) on Ca^2+^-transients elicited in astrocytes by the stimulation of synaptic pathways (Figure 2). We used short burst of high-frequency stimulation (see Methods) to mimic the conditions of elevated neuronal activity which can occur either in the physiologic context or during induction of long-term synaptic potentiation. Ca^2+^-signaling was triggered in the CA1 astrocytes by the activation of Schaffer collaterals and in the layer 2/3 astrocytes by the activation of intracortical afferents. We have shown previously that major components of synaptically-induced astrocytic Ca^2+^-activity are mediated by NMDA, mGluR, P2X, P2Y and endocannabinoid receptors (Palygin et al., 2010; Rasooli-Nejad et al., 2014; Pankratov and Lalo, 2015; Lalo et al., 2019; Lalo and Pankratov, 2022).

We also evoked Ca^2+^ -signaling with agonists of PAR1, adrenergic α1 and NMDA receptors, which were previously reported to bring significant contribution into astrocytic signaling and activate release of gliotransmitters (Palygin et al., 2010; Lalo et al., 2014; Pankratov and Lalo, 2015). In terms of Ca^2+^-signaling, the agonists of PAR1 and α1-adrenoreceptors, correspondingly TFLLR and NA, have been shown to act specifically in the astrocytes so they are widely used as a tool to activate astrocyte-driven modulation of synaptic plasticity (Woo et al., 2012; Lalo et al., 2014; Pankratov and Lalo, 2015; Won et al., 2021; Koh et al., 2022).

In line with our previous reports, the HFS activated the robust Ca^2+^-response in the wild-type and dnSNARE mice (Figures 2A, B) and significantly increased the amplitude and frequency of spontaneous Ca^2+^-transients in the astrocytic microdomains (Figures 2A, C). Application of ryanodine did not cause notable effect on the baseline Ca^2+^-level and spontaneous signaling in astrocytes but strongly suppressed the HFS-induced response and facilitatory action of HFS on Ca^2+^-transients in the microdomains (Figures 2A–C). Similar to HFS, the 30 s-long bath application of agonist of astrocytic receptors caused a notable Ca^2+^-elevation accompanied by the increase in the spontaneous Ca^2+^-transients. Again, similar to the effects of HFS, the effects of TFLLR, NA and NMDA on astrocytic signaling were efficiently blocked by the ryanodine.

The astrocytes of the old WT mice exhibited the decrease in the basal amplitude and frequency of spontaneous Ca^2+^-transients but retained responsiveness to stimulation (Figures 2D, F). Similar to the adult mice, the HFS, TFLLR, NA and NMDA produced notable effect on the Ca^2+^-signaling in the astrocyte of old mice, which was dramatically reduced by ryanodine. In both age groups, there was no significant difference in the effects of stimulation and ryanodine in the astrocytes of dnSNARE mice (*n* = 7 for adult and 6 for old mice) and their wild-type counterparts (the data are not shown).

**FIGURE 3:**
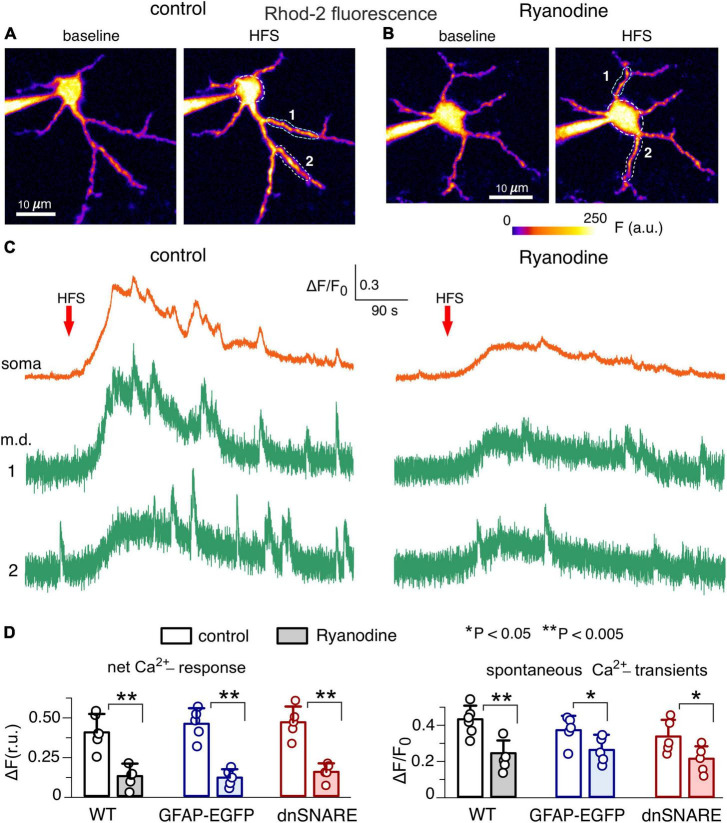
Impact of intracellular ryanodine on Ca^2+^-signaling in the hippocampal astrocytes. **(A,B)** Representative multi-photon presudo-color images of Rhod-2 fluorescence recorded in the CA1 astrocytes of adult dnSNARE mice perfused with 20 μM Rhod-2 **(A)** and Rhod-2 and 10 μM ryanodine. **(B)** Images were acquired before (baseline) and after the short train of high-frequency stimulation of CA3-CA1 synapses (HFS). **(C)** Examples of Ca^2+^-transients recorded in the soma and microdomain (m.d.) ROIs in control (left) and in the presence of intracellular M ryanodine (right) are shown below. Note the marked increase in the spontaneous Ca^2+^-elevations after HFS in control and inhibition of the HFS-induced signaling by the intracellular ryanodine. **(D)** The pooled data on the net cell response to HFS and amplitude of the spontaneous Ca^2+^-transients recorded in the CA1 astrocytes of the wild-type, GFAP-EGFP and dnSNARE mice. Dots indicate individual experiments. Asterisks (*,**) indicate the statistical significance of the effects of ryanodine.

One should note that application of exogenous ryanodine could also affect neuronal RyRs which might affect the release of neurotransmitters and, theoretically, alter neuron-to-astrocyte communication. However, the main action of neuronal RyRs would be augmentation of Ca^2+^-influx via voltage-gate Ca^2+^-channels and NMDA receptors which would require significant depolarization and thereby could manifest at high levels of synaptic activity. Therefore, putative inhibition of neuronal RyRs could not strongly affect the above responses of astrocytes to TFLLR, NA and NMDA since they were recorded in the presence of TTX (see Methods). We have also shown that primary effect of application of these compounds in the presence of TTX is activation of astrocytic Ca^2+^-signaling. However, one could not *a priori* rule out putative influence of neuronal RyRs on the astrocytic signaling evoked by HFS. To dissect the specific contribution of astrocytic RyRs, we evaluated the responses of astrocytes to HFS under conditions of intracellular perfusion with 10 μM ryanodine (Figure 3). In addition to the dnSNARE mice and their wild-type littermates, we also used the transgenic mice expressing enhanced green fluorescent protein (EGFP) under the control of the glial fibrillary acidic protein (GFAP) promoter; usage GFAP-EGFP mice facilitated identification of astrocytes (Palygin et al., 2010; Lalo et al., 2011). In the adult mice of all three genotypes, the net Ca^2+^-response of CA1 astrocytes perfused with ryanodine and Rhod-2 was significantly reduced in the comparison to their counterparts perfused only with Rhod-2 (Figures 3B, C). Intracellular ryanodine also significantly decreased the amplitude of the spontaneous Ca^2+^-transients in the hippocampal astrocytes (Figures 3C, D). These results closely agree with the data obtained using exogenous application of ryanodine (Figure 2) and allow to rule out the impact of neuronal RyRs on the astrocytic Ca^2+^-signaling, at least in our experimental setting.

Thus, our data show that RyR-mediated Ca^2+^-induced Ca^2+^-release from ER brings substantial contribution into signaling in the hippocampal and neocortical astrocytes and, potentially, can play instrumental role in the release of gliotransmitters. This notion was tested in the following experiments.

### Impact of ryanodine receptor modulators on release of gliotransmitters and glia-to-neuron communications

**FIGURE 4:**
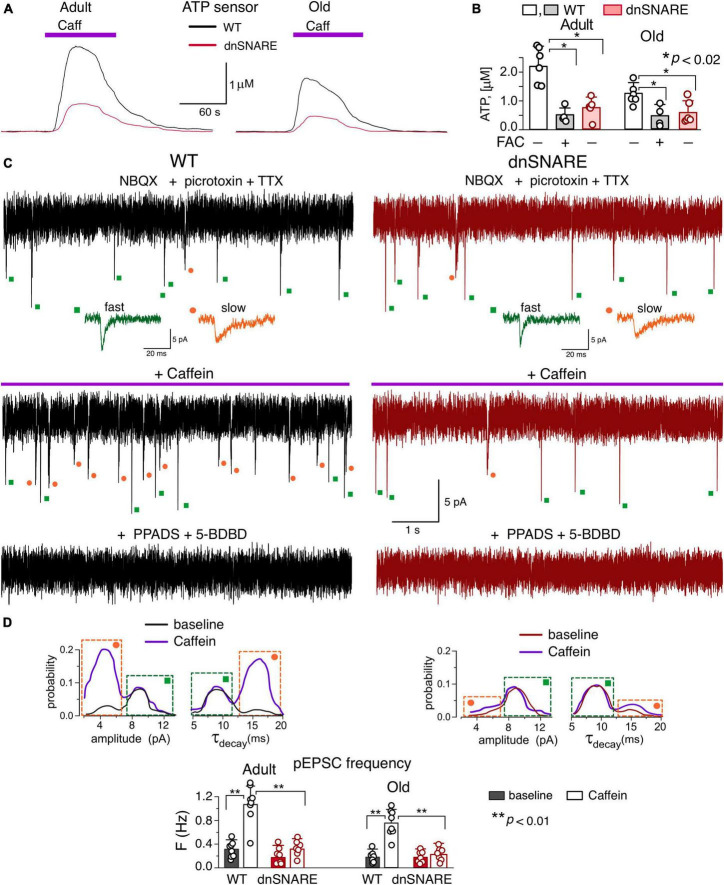
Caffein-activated release of ATP in hippocampal slices. **(A,B)** Release of ATP assessed with microelectrode biosensors to ATP placed in the *stratum radiatum* of CA1 area of hippocampus of WT and dnSNARE mice of two age groups. **(A)** Representative ATP-responses to the application of caffein. **(B)** The average peak amplitude of caffein-elicited ATP-transients recorded in the adult and old mice in the control and after incubation of slices with glia metabolic poison FAC (mean ± SD for number of slice indicated by dots); asterisks (*) indicate the significance of difference between genotypes and between control and FAC for WT mice. **(C–E)** Spontaneous purinergic currents were recorded in the CA1 pyramidal neurons of WT (left) and dnSNARE(right) mice at –80 mV in the presence of picrotoxin, NBQX, TTX, DPCPX and SCH58261. **(C)**, top to bottom: the whole-cell currents recorded in the baseline, 3 min after start of 5 min-long caffein application, and after the second caffein application in the presence of P2X receptor antagonists PPADS and 5-BDBD (25 min after the first one). The dots indicate correspondingly the purinergic events of fast (green) and slow (orange) kinetics; the examples of individual events are shown in the inlays. Note the significant increase in the number of slow events during caffein application and elimination of all transient currents by purinergic antagonists. **(D)** The amplitude and decay time (τ_decay_) distributions of the spontaneous purinergic currents (pEPSCs) recoded before (baseline) and within 2–10 min after application of caffein. Note the increase in the fraction of events of smaller quantal amplitude (left peak) and slower decay (right peak in the τ_decay_ histogram) in the WT mice after application of caffein. **(E)**, the average frequency of smaller-and-slower purinergic currents (identified as shown in panels **C,D**) in the CA1 neurons of WT and dnSNARE mice before (baseline) and after application of caffein (in 2–10 min window); mean ± SD for the individual experiments indicated by dots. Asterisks (**) indicate the significance of difference from the baseline and between the genotypes.

To verify the participation of the RyRs in the gliotransmission, we evaluated the caffeine-induced release of ATP in the CA1 area of brain slices of wild-type and dnSNARE mice. The extracellular ATP was detected with the aid of microelectrode biosensors, as described previously (Frenguelli et al., 2007; Gourine et al., 2010; Lalo et al., 2014; Rasooli-Nejad et al., 2014). Both in the adult and old mice, activation of CA1 astrocytes by 20 μM caffeine (in the presence of A1 and A2 receptors antagonists) caused the transient elevation in the extracellular ATP (Figure 4A) which was strongly suppressed in the dnSNARE mice. In comparison to their wild-type counterparts (Figures 4A, B), the caffeine-induced ATP-transients were reduced in the adult and old dnSNARE-expressing mice correspondingly by 72 ± 18% and 79 ± 16% (Figure 4B). Also, the caffeine-evoked ATP-transients in the CA1 area of WT mice were dramatically inhibited after 20 min-long incubation of brain slices with glial metabolic poison fluoroacetate (FAC, 2mM) (Figure 4B). These observations strongly suggest that caffeine-induced ATP release originated from astroglial exocytosis.

Then, we verified that caffeine-induced exocytosis of ATP from astrocytes can activate neuronal purinoreceptors. The hippocampal and neocortical pyramidal neurons, by virtue of expression of P2X receptors ( Pankratov et al., 1998, 2007), can act as a natural *in situ* ATP-sensors. We have previously shown that release of ATP from astrocytes can activate transient transmembrane currents in the CA1 and layer 2/3 neurons which can be distinguished from the purinergic currents of synaptic origin by their slower kinetics and smaller amplitude (Pankratov et al., 1998; Lalo et al., 2014, 2016).

To detect the putative astrocyte-derived purinergic activity, excitatory whole-cell currents were recorded in the CA1 pyramidal neurons at a membrane potential of −80 mV in the presence of TTX (1 μM) and inhibitors of A1, A2 receptors. For pharmacological isolation of purinergic currents, NBQX (30 μM), D-APV (30 μM) and picrotoxin (100 μM) have also been applied to eliminate the synaptic signaling via AMPA, NMDA and GABAA receptors. Similar to our previous reports (Pankratov et al., 1998, 2007; Lalo et al., 2016), we detected the non-glutamatergic excitatory spontaneous currents which were fully inhibited by application of P2X receptor antagonists PPADS (10 μM) and 5-BDBD (5 μM) in all 15 neurons tested (Figure 4C).

Under baseline conditions, the purinergic spontaneous currents recorded in the CA1 neurons of adult WT mice had the mean amplitude of 8.9 ± 3.2 pA and decay time of 8.9 ± 2.9 ms (*n* = 8). After bath application of caffeine, the frequency of the purinergic spontaneous currents underwent a dramatic increase in the neurons of wild-type mice (Figures 4C, E) which was paralleled by the reduction of their mean amplitude to 6.7 ± 1.7 pA and slowing down of their decay kinetics.

Such changes in the average amplitude and decay time of purinergic currents were caused by occurrence of large number of spontaneous events of smaller amplitude and slower kinetics, as it was evidenced by appearance of two distinct peaks in their amplitude and decay time distributions (Figure 4D). This result is consistent with our previous experiments where bimodal distributions of amplitude and decay time of spontaneous purinergic currents were observed in the neocortical (Lalo et al., 2014; Rasooli-Nejad et al., 2014; Pankratov and Lalo, 2015) and hippocampal (Lalo and Pankratov, 2022) neurons. Our previous experiments have proved that purinergic currents with smaller amplitude and large decay time originate from the astrocytic exocytosis of ATP (Lalo et al., 2014).

Application of caffein caused the significant increase in the number of these smaller and slower currents (Figures 4C, D). The caffein-evoked burst of purinergic EPSCs (pEPSCs) in the CA1 neurons was dramatically inhibited in the dn-SNARE mice, strongly supporting their origin from astroglial exocytosis (Figures 4D, E).

**FIGURE 5:**
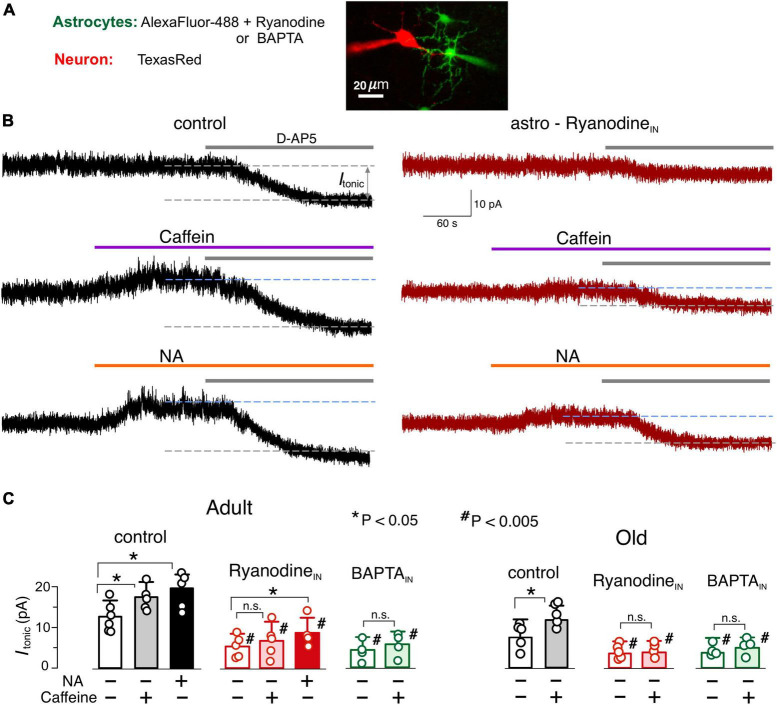
Astrocyte-driven modulation of tonic NMDA receptor-mediated currents in neocortical neurons. **(A)** The NMDA receptor-mediated transmembrane currents were recorded in the layer 2/3 pyramidal neuron with whole-cell pipette filled with TexasRed fluorescent dye. Simultaneously, a nearby astrocyte was perfused with intracellular solution containing either AlexFluor488 fluorescent dye alone (as a control), or AlexaFluor488 + ryanodine (10 μM) or AlexaFluor488 + BAPTA (3 mM). The tonic NMDAR-mediate currents were assessed 10–15 min after established staining of neighboring astrocytes. **(B)** The examples of NMDAR-currents recorded in the pyramidal neurons of adult WT mice in the control (left column) and during intracellular perfusion of astrocytes with ryanodine (right column). The magnitude of tonic NMDAR-currents was evaluated by the shift in the whole-cell holding current (I_tonic_) caused by the application of D-APV (50 μM). Currents were measured in presence of NBQX (50 μM), and DPCPX (3 μM) and SCH58261 (1 μM) at holding potential of +40 mV. D-APV was applied either alone (baseline, upper traces) or after activation of astrocytic signaling by 20 μM caffein (middle traces) or 3 μM noradrenaline. **(C)** The average amplitude of tonic NMDAR-current measured in the mice of two age groups under different conditions of astrocyte perfusion; the data are shown as mean ± SD for the individual cell indicated by dots. Asterisks (*,**) correspondingly indicate statistical significance of the effects of caffein and NA on tonic current as compared to the baseline (i.e., without activation of astrocytes). The hush symbols (#) indicate statistical significance of the effect of astrocytes perfusion with ryanodine and BAPTA as compared to the same agonist (caffein or NA) in control. Note the enhancement of the tonic current caused by caffein and NA and strong attenuation of the tonic current with astrocytic ryanodine.

The putative mechanisms of Ca^2+^-dependent astroglial release of glutamatergic gliotransmitters, Glutamate and D-Serine, are still under intensive study and debate (Lalo et al., 2021). Recent studies provided strong evidence supporting both fast exocytosis (Won et al., 2021; de Ceglia et al., 2023) and slow channel-mediated release (Woo et al., 2012; Koh et al., 2022). The most plausible target of glutamate released by astrocytes is neuronal NMDA receptors which have high affinity to glutamate and are abundantly expressed both at synaptic densities and extrasynaptic sites (Paoletti et al., 2013; Sanz-Clemente et al., 2013; Papouin and Oliet, 2014). One of the prominent effects of glia-derived glutamatergic transmitters on neuronal NMDA receptors is their tonic activation, whose origin from Ca^2+^-dependent release of glutamate and D-Serine has been recently verified by Koh et al. (2022). Thus, we measured the tonic NMDA current in the neocortical pyramidal neuron as a readout of glutamatergic gliotransmission (Figure 5).

To assess the neuronal response to glia-derived glutamate we recorded the whole-cell transmembrane NMDAR-mediated currents in neocortical pyramidal neurons at membrane potential of + 40 mV and physiological Mg^2+^concentration (Figure 5). Activity of AMPA, GABA and P2X receptors was blocked by DNQX, picrotoxin and PPADS plus 5-BDBD. The amplitude of NMDAR-mediated tonic current was elevated by the downward shift in the holding current caused by application of NMDAR antagonist D-AP5 (3 μM). The selective activation of Ca^2+^-signaling in astrocytes (Woo et al., 2012; Lalo et al., 2014; Pankratov and Lalo, 2015) was achieved with a 30 sec-long application of 3 μM noradrenaline or 20 μM caffein (as in Figure 1).

To verify that astrocytes regulate the neuronal NMDAR tone, the tonic NMDAR-current was measured in the pyramidal neurons while Ca^2+^ signaling in nearby astrocytes was inhibited either by Ca^2+^-clamping or intracellular ryanodine. For attenuation of Ca^2+^-signaling, astrocytes were perfused via the patch-pipette with intracellular solution containing fluorescent dye AlexaFluor488 and 3 mM BAPTA or 10 μM ryanodine; as a control condition, loading of astrocytes with AlexaFluor488 only was used (Figures 5A, C).

The NMDAR tonic current recorded in the WT mice under basal conditions (without astrocytes activation) reached 13.2 ± 4.1 pA (*n* = 6) and 9.4 ± 3.5 pA (*n* = 5) at adult and old age correspondingly (Figure 5C). Activation of astrocytes with NA or caffein substantially increased the tonic current in both age groups with NA producing larger effect (Figure 4A). The NA- and caffein-induced elevation in the tonic NMDAR-current was significantly inhibited by perfusion of astrocyte both with BAPTA and ryanodine.

Taken together, the above results indicate that RyR-mediated CICR is instrumental for Ca^2+^- dependent release of ATP and glutamate from astrocytes. Both exocytosis of ATP and glial modulation of NMDAR-tone have been implicated in astroglial control of synaptic plasticity and memory (Lalo et al., 2016; Koh et al., 2022; Lalo and Pankratov, 2023). So, one might expect an involvement of astrocytic RyRs in regulation of neuronal plasticity in neocortex and hippocampus.

### Impact of astrocytic ryanodine receptors on glial modulation of long-term synaptic plasticity

**FIGURE 6:**
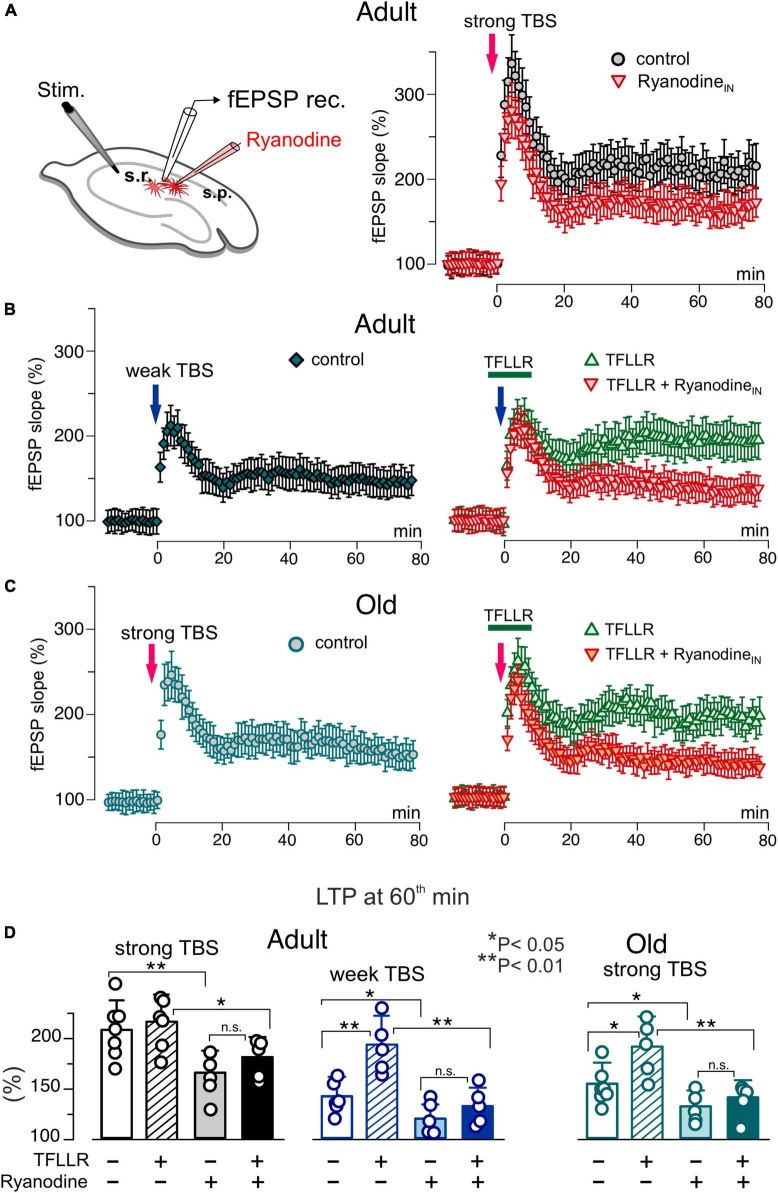
Impact of astrocytic ryanodine receptors on long-term synaptic plasticity in CA1 hippocampal area. **(A)** The LTP of the field EPSPs was induced in the CA1 hippocampal area by 5 (weak) or 10 (strong) trains of theta-burst stimulation (TBS) as described in Materials and methods. The LTP was measured in the wild-type mice of two age groups under different conditions: control, after intracellular perfusion of astrocyts, lying in the vicinity of recording site, with ryanodine (similar to the Figure 4) and application PAR-1 agonist TFLLR for 5 min prior and 5 min after delivering TBS either on its own or after perfusion of astrocytes with ryanodine. **(A–C)** The time course of changes in the slope of fEPSPs recorded under different conditions. Dots in the graphs represent the average of 6 consecutive fEPSPs; data are shown as mean ± SD for 5–7 experiments (as indicated in panel **D**). Data were normalized to the fEPSP slope averaged over 10 min period prior to the TBS. **(A)** Inhibition of the RyRs in astrocytes significantly reduced the magnitude of LTP induced by the strong TBS in the adult mice. **(B)** Changes in the fEPSPs induced in the adult mice by the weak TBS; the magnitude of LTP was much smaller as compared to the result of strong TBS (see panel **A**). Activation of astrocytes with TFLLR significantly the enhanced the LTP which was prevented by the inhibition of astrocytic RyRs. **(C)** The strong TBS-induce LTP exhibits decreased significantly in the old mice. Activation of astrocytes with TFLLR rescued the LTP; the effect of TFFLR was strongly attenuated by the inhibition of astrocytic RyRs. **(C)** The pooled data on the magnitude of the CA1 LTP under different conditions evaluated as relative increase in the fEPSP slope at 60 min, averaged across 10 min time window. Data are shown as mean ± SD for the individual experiments indicated as dots. The asterisks (*,**) indicated statistical significance (2-population unpaired *t*-test) for the difference between treatment groups. Note the significant decrease in the LTP magnitude caused by inhibition of astrocytic RyRs.

**FIGURE 7:**
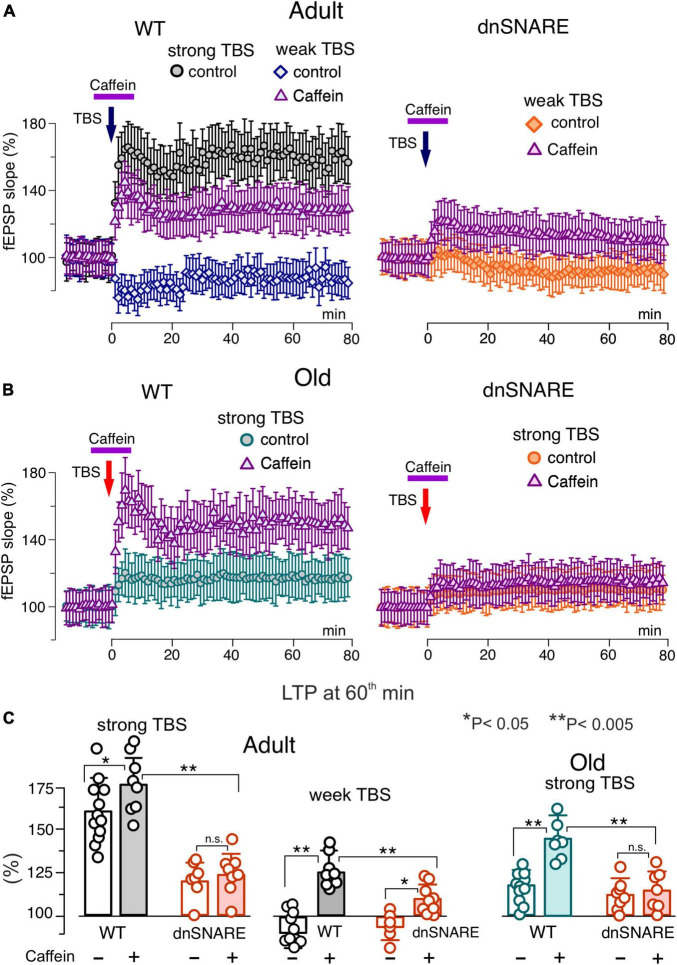
Impact of caffein on synaptic plasticity in the neocortex. The long-term potentiation of fEPSPs in the neocortical layer 2/3 was induced by 20 (weak) or 50 (strong) trains of theta-burst stimulation (TBS) in the wild-type and dnSNARE mice either in control or after application of caffein 5 min prior and 5 min after delivering TBS (in the presence of A1 and A2 receptor antagonists). **(A,B)** The time course of changes in the slope of fEPSPs recorded under different conditions. Dots in the graphs represent the average of 6 consecutive fEPSPs; data are shown as mean ± SD for 6–12 experiments (as indicated in panel **C**). Data were normalized to the fEPSP slope averaged over 10 min period prior to the TBS. **(A)** In the adult mice under control condition, the strong TBS induced strong LTP but weak TBS did not. Application of caffein enables the weak TBS to induce LTP in the wild-type but in the dnSNARE mice. **(B)** The magnitude of LTP induced by the strong TBS was significantly reduced in old mice in the control but increased substantially when induced after application of caffein. The effect of caffein was abolished in the dnSNARE mice. **(C)** Pooled data on the magnitude of LTP evaluated as relative increase in the fEPSP slope at 60th min, averaged across 10 min time window. Each data point shows mean ± SD for individual experiments indicated as dots. Asterisks (*,**) indicate statistical significance of difference in the LTP magnitude between control and caffein and between genotypes (unpaired *t*-test). Note the significant changes in LTP caused by caffein in the wild-type but not the dnSNARE mice.

We assessed the long-term potentiation (LTP) of the field EPSPs in the CA1 hippocampal area and layer II/III of somatosensory cortex of the wild-type mice of two age groups (Figures 6, 7). In the first series of experiments, we used intracellular perfusion of astrocytes with ryanodine and Alexa488 in the vicinity of fEPSP-recording electrode similarly to the experiments described in the Figure 5. As a control condition, a perfusion of astrocytes with Alexa488 alone was used.

As we showed previously, a modulatory role for astrocytes manifested prominently in facilitation of LTP induction by sub-threshold high-frequency stimulation (Pankratov and Lalo, 2015; Lalo et al., 2018). So, we used short and long trains of theta-burst high-frequency stimulation further referred for simplicity as “weak TBS” and “strong TBS”; details of induction protocols for CA1 and L2/3 areas are given in the Methods. In the CA1 area of adult mice, the weak TBS induced notable increase in the fEPSP slope only in 3 of 6 trials, with the average potentiation at 60^th^ min reaching only 44 ± 26% (above the baseline) whereas the strong TBS induced robust the LTP amounting to 105 ± 26% (Figures 6A–C). In the old mice, the weak TBS was not able to induce any notable LTP (*n* = 5, data not shown) and the magnitude of LTP induced by the strong TBS was significantly reduced as compared to the adult mice (Figures 6C, D). The activation of astrocytes with TFLLR during the induction enabled the weak TBS to induce the substantial LTP in both age groups. The facilitatory effect of TFLLR on LTP induced by the strong stimulus was statistically significant only in the old mice. When fEPSPs were recorded in the vicinity of CA1 astrocytes perfused with ryanodine, the weak TBS did not cause any marked potentiation and the magnitude of the LTP induced by strong TBS decreased considerably (Figures 6A, D). Importantly, the inhibition of astrocytic RyRs strongly attenuated the facilitatory effect of astrocytes activation (Figure 6D) in both age groups.

In the second series of experiments, we explored the effects of caffein on the LTP in the neocortex of wild-type and dnSNARE mice (Figure 7). In line with our previous results, the LTP registered in the layer 2/3 of somatosensory cortex of wild-type mice under control conditions exhibited sharp stimulus- and age-dependence. In particular, the weak TBS induced a moderate long-term depression of fEPSPs and the magnitude of LTP was substantially lower in the old mice (Figures 7A, B) as compared to their younger counterparts. In the wild type mice, application of caffein during the LTP induction led to dramatic changes in the effect of weak TBS, switching it to the long-term potentiation in all trials (125 ± 22%, *n* = 5). Caffein also increased the magnitude of LTP induced by the strong TBS with a prominent effect in the old mice (Figure 7B). The facilitatory effect of caffein was abolished in the dnSNARE mice (Figures 7B, C), strongly supporting an involvement of gliotransmission in this effect.

Combined, these data imply that astroglial ryanodine receptors contribute to Ca^2+^-dependent release of gliotransmitters and, thereby, into astrocyte-driven modulation of synaptic plasticity in the hippocampus and neocortex.

## Discussion

Combined, our data suggest a prominent role for RyRs in the astroglial function across a lifetime which manifests in the augmentation of intracellular Ca^2+^-signaling (Figures 1, 2) both in the astrocytic somata and functional microdomains. Our results agree with recent reports on participation of RyRs and CICR in spontaneous and neurotransmitter-evoked signaling (Rodriguez-Prados et al., 2020; Skowronska et al., 2020; Weiss et al., 2022) and previous data on expression of RyRs (predominantly of type2 and 3) in astrocytes of different brain regions (Martone et al., 1997; Simpson et al., 1998; Matyash et al., 2002; Kovacs et al., 2021). Our data also indicate a certain decline in the activity of RyRs during (non-pathological) aging which contrasts with observations of upregulations of RyRs expression in some pathologies (Kesherwani and Agrawal, 2012; Kovacs et al., 2021).

As one could expect, RyR-mediated Ca^2+^-release was independent on the initial source of Ca^2+^-and caused enhancement of astrocytic signaling triggered by a wide range of GPCRs and ionotropic receptors (Figure 2). Hence, RyR-mediated CICR can potentially be implicated in the great variety of pathways of glia-neuron communications. In the present work, we have shown just few examples of involvement of ryanodine receptors in regulatory cascades (Figures 5, 6) whose role in the astroglial modulation of synaptic transmission plasticity was rather well-documented (Rasooli-Nejad et al., 2014; Pankratov and Lalo, 2015; Lalo et al., 2018, 2021; Durkee and Araque, 2019; Koh et al., 2022). It is conceivable that CICR can be involved in many other mechanisms of astrocyte-neuron interactions which are yet to be explored.

There is an important notion arising from our observations that caffein, can trigger release of gliotransmitters (Figures 3, 4) which in turn can enhance the LTP in the old animals (Lalo et al., 2018, 2021; Lalo and Pankratov, 2023). We would like to emphasize that these effects of caffein were not related to the inhibition of A1 and A2 receptors and occurred via elevation of cytosolic Ca^2+^-level in astrocytes. Also, our results confirmed that expression of dnSNARE transgene in astrocytes did not affect the Ca^2+^-induced Ca^2+^-release from ER, so difference in the putative effects of caffeine in the WT and dnSNARE mice should be attributed to its action on gliotransmission.

As our data indicate (Figures 6, 7), the RyR-related effects of caffein can counter-balance the age-related decline in the synaptic plasticity, acting independently of A1 receptors (Figure 7). We would like to note that although the participation of neuronal RyRs in the beneficial effect of caffein on LTP cannot be excluded, significant attenuation of this effect both in the dnSNARE mice (Figure 7) and after intracellular perfusions of astrocytes with ryanodine (Figure 6) strongly suggests the substantial contribution of astrocytic RyRs. Interestingly, beneficial effects of caffein on cognitive function in elderly individual are rather well-documented. There is a number of epidemiological studies reporting a negative correlation between that regular caffein consumption and progression of cognitive decline or risk of developing AD and dementia later in life (Eskelinen and Kivipelto, 2010; Santos et al., 2010; Barberger-Gateau et al., 2013). Association of caffein consumption with lower risk of stroke and dementia have been recently confirmed with the large cohort data (Zhang et al., 2021). In the mouse models of AD, oral administration of caffein was reported to ameliorate decline in learning and memory and reduce β-amyloid tissue and plasma concentrations and neuronal loss (Arendash et al., 2006; Laurent et al., 2014; Yelanchezian et al., 2022; Stazi et al., 2023; Tiwari et al., 2023). Beneficial effects of caffein on cerebral vascularity and blood flow have also been reported both in animal models and human patients (Chen et al., 2008; Gaspar et al., 2024).

Historically, the cognitive effects of caffein were usually ascribed to the inhibition of adenosine receptors (Cellai et al., 2018; Yelanchezian et al., 2022; Merighi et al., 2023) and importance of caffein action as CICR-activator in the brain cells was often overlooked. However, there is a growing evidence of neuroprotective and cognitive effects of caffein which cannot be directly attributed to the inhibition of A1/A2 receptors (Yelanchezian et al., 2022). For instance, caffein administration has been reported to improve memory via facilitation of neuronal progenitor cell survival (Tiwari et al., 2023) and ameliorate neuroinflammation and neurodegeneration in the hippocampus in microglia- and astrocyte-dependent manner (Tiwari et al., 2023). Also, caffein has been reported to ameliorate some forms of pathological cognitive dysfunction acting via ryanodine receptors (Liu et al., 2023).

An importance of astrocytes in mediating the neurovascular interface and orchestrating neuroprotection is widely recognized (Giaume et al., 2010; Halassa and Haydon, 2010; Liu et al., 2017; Verkhratsky and Nedergaard, 2018; Verkhratsky et al., 2019) and a key role for Ca^2+^-signaling in homeostatic functions of glia cells is well established (Halassa and Haydon, 2010; Bazargani and Attwell, 2016; Verkhratsky and Nedergaard, 2018). It is conceivable, therefore, that neuroprotective effects of caffeine could, at least partially, result from its action as activator of astrocytic ryanodine receptors. Thus, role for caffein-RyR-astrocyte axis in cognitive enhancement at the old age and neurodegenerative disorders is worthwhile the further investigation.

To conclude, our data demonstrate that ryanodine receptors participate in the activation of hippocampal and neocortical astrocytes leading to release of gliotransmitters which, in turn, activates postsynaptic purinergic and glutamatergic signaling in the neighboring neurons. This signaling cascade contributes to astrocytic control of synaptic plasticity and might underlie, at least partially, neuroprotective, and cognitive effects of caffein.

## Data availability statement

The datasets presented in this study can be found in online repositories. The names of the repository/repositories and accession number(s) can be found below: doi: 10.6084/m9.figshare.20496516.

## Ethics statement

The animal study was approved by the University of Warwick Animal Welfare and Ethical Review Body (AWERB). The study was conducted in accordance with the local legislation and institutional requirements.

## Author contributions

UL: Conceptualization, Data curation, Formal analysis, Investigation, Methodology, Writing – original draft. YP: Conceptualization, Data curation, Formal analysis, Funding acquisition, Investigation, Methodology, Project administration, Supervision, Writing – original draft, Writing – review and editing.
